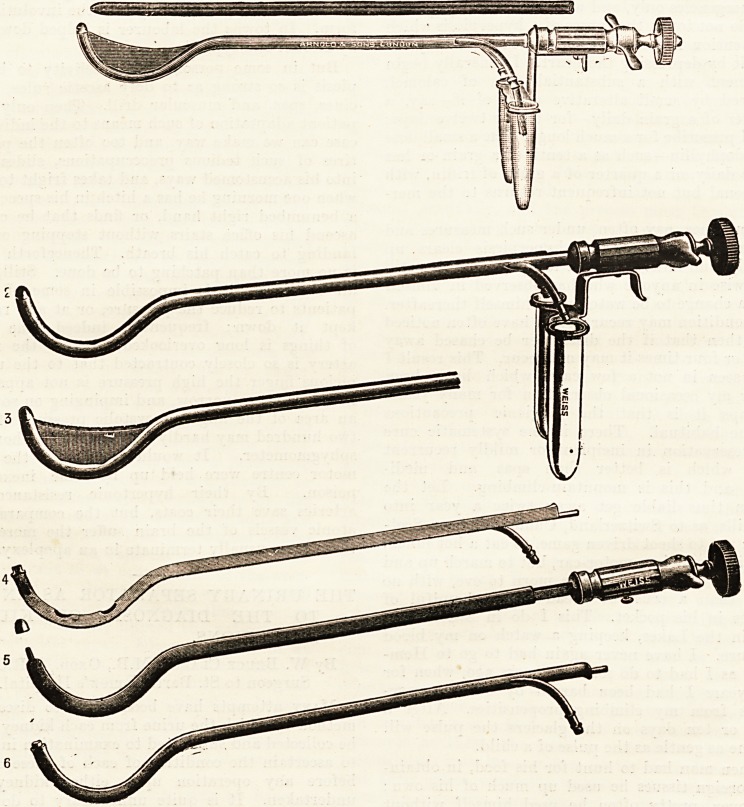# The Urinary Separator as an Aid to the Diagnosis of Kidney Affections

**Published:** 1905-10-14

**Authors:** W. Bruce Clarke

**Affiliations:** Surgeon to St. Bartholomew's Hospital


### THE URINARY SEPARATOR AS AN AID TO THE DIAGNOSIS OF KIDNEY AFFECTIONS.

By W. Bruce Clarke, M.B., Oxon., F.R.C.S.,
Surgeon to St. Bartholomew's Hospital.

Many attempts have been made to discover a method by which the urine from each kidney could be collected and submitted to examination in order to ascertain the condition of each of these organs before any operation upon either kidney was undertaken. It is quite unnecessary to do more than allude to these attempts. In some cases catheterisation of the ureters has been undertaken, but the procedure demands a large amount of skill and practice in the operator, and, even in trained hands, is very often attended by complete failure.

A method which is uncertain is worse than useless, because it is liable to mislead.

Quite recently, however, an instrument has been devised to which none of these objections apply. It is simple to introduce, practically infallible in action, innocuous to the patient, and does not demand an anæsthetic.

The Luys separator which is shown in fig. 1 converts the bladder into two halves for the time being by means of a vertical septum. The result is that each lower half of the bladder receives the urine from its corresponding ureter, and delivers it by a separate channel into a glass tube on its own side.

Fig. 2 shows the instrument when it is ready for introduction. It is shaped somewhat like an ordinary male catheter. It consists of a middle portion (shown in fig. 5), to each side of which is attached a small catheter (figs. 4 and 6).

In fig. 2 these small catheters are shown fixed to the middle portion with their ends leading into two collecting tubes.

When the instrument is required to be introduced the middle portion is covered by a rubber sheath (fig. 1 (*a*)), which slides over and fits tight to it.

Lying in the concavity of the instrument, and therefore not seen in any of these figures, is a small chain, much of the same construction as a miniature bicycle chain. This chain is actuated by a screw in the handle, which makes it lie at will either loosely in the semicircular groove between the two catheters, or causes it to stretch tightly across the semicircle. It is placed in this latter position in figs. 1 and 3, but is invisible because it is covered by the rubber sheath, which has been stretched by the tightening of the chain and is thus for the time being converted into a vertical septum.

When the separator is introduced into the patient's bladder the chain lies along its circumference. As soon as the chain is tightened it forms the diameter of the semicircle, and the rubber sheath is stretched and divides the bladder into two halves.

The instrument is available for both sexes. When it is introduced the patient sits up either in bed or on a chair, so as to prevent the water from the two kidneys from flowing over the septum, which almost invariably occurs if the patient is in a recumbent position.

It may be necessary to carry on one's observations for more than half an hour before a suitable amount of urine has been collected from the two kidneys, especially if one of the organs is in a damaged condition, and therefore secreting at a slower rate than is usually the case.

It is also possible by the aid of the separator to ascertain the permeability of each kidney to certain substances which can readily be detected in the urine. Methylene blue is very commonly employed for this purpose, and is usually administered in the form of a 3-grain pill, or the same amount is enclosed in a gelatine capsule. If the kidneys are healthy, methylene blue will be secreted with the urine and perceptibly colour it in half an hour or less. But if one kidney is affected by disease and the other is healthy, it may be as much as eighteen hours before the methylene blue is secreted by the diseased kidney.

The amount of urea and other urinary salts will also be found to be considerably diminished in the diseased kidney, whilst it is obvious that pus and blood, if they are flowing from one kidney only, will be readily detected by ocular inspection.

A few instances will indicate the uses to which such an instrument may be put with advantage. The question is often asked, Does a kidney in a condition of hydronephrosis, provided it is painless, demand operative interference? The separator affords a ready answer to this question, as the following case will show. The patient in question had an obvious left hydronephrosis, and, beyond a little discomfort if the kidney got more than usually distended, suffered no annoyance whatever. The separator was introduced on several occasions. On the first two or three ocasions when it was employed no urine flowed down the left ureter. As the patient was generally made aware by her own sensations when the urinary flow had been re-established after a period of temporary distension, an opportunity was taken to introduce the separator when the hydronephrosis was in the process of emptying itself.

Urine passed in a continuous stream, but only from the left side, during the twenty minutes of the observation. The healthy kidney, which, no doubt, had been doing its best to supplement the work of its diseased neighbour, was for the moment taking a rest. But if the quantity of urine which was issuing from the left side was abundant, it was deficient in solid contents, and contained less than half the amount that had been previously registered as coming from the right side.

The kidney, which had been previously fixed by operation, was a few days later cut down upon and refixed much higher in the lumbar region, so as to avoid any further kink in the ureter. The hydronephrotic distension did not recur, and the urine from the left side was found a few weeks later to contain its normal proportion of salts.

There are no cases in which the value of this instrument is so clearly seen as in those of obstructive suppression—namely, where one kidney has been completely destroyed by a calculus, and where complete obstruction is threatened owing to the impending blockage of the opposite ureter. There may be, and often is, but little indication afforded by the symptoms as to which is the better of the two kidneys, but the separator sets the matter at rest at once, as the following instance shows. When the urine was collected through the separator its appearance on the two sides was absolutely different. That from the right side was small in amount (scarcely a teaspoonful), whilst more than three-quarters of an ounce had come from the left kidney. The chemical examination was even more convincing. The urine of the right kidney was alkaline and contained albumen and pus. That from the left was acid clear and exhibited not a trace of albumen or pus. The inference was that the kidney on the left side was the working one, but that it contained presumably a calculus. It was accordingly submitted to operation, and a stone removed from its pelvis.

Just as the perfecting of the cytoscope has enabled us to diagnose with certainty what the manipulations of the bladder-sound could never lead us to infer, so the separator has rendered possible the collection of the urine from each kidney separately, and certainly enables us to find the solution of many kidney troubles that were before insoluble.

## Figures and Tables

**Figure f1:**